# Reproducibility of the principal component analysis (PCA)‐based data‐driven respiratory gating on texture features in non‑small cell lung cancer patients with ^18^F‑FDG PET/CT

**DOI:** 10.1002/acm2.13967

**Published:** 2023-03-21

**Authors:** Shohei Fukai, Hiromitsu Daisaki, Mitsutomi Ishiyama, Naoki Shimada, Takuro Umeda, Kazuki Motegi, Ryoma Ito, Takashi Terauchi

**Affiliations:** ^1^ Department of Nuclear Medicine The Cancer Institute Hospital of Japanese Foundation for Cancer Research Tokyo Japan; ^2^ Graduate School of Radiological Technology Gunma Prefectural College of Health Sciences Gunma Japan; ^3^ Graduate School of Medical and Dental Sciences Tokyo Medical and Dental University Tokyo Japan

**Keywords:** 18F‐FDG, data‑driven respiratory gating, image feature, non‑small cell lung cancer, PET/CT, texture analysis, texture feature

## Abstract

**Objective:**

Texture analysis is one of the lung cancer countermeasures in the field of radiomics. Even though image quality affects texture features, the reproducibility of principal component analysis (PCA)‐based data‑driven respiratory gating (DDG) on texture features remains poorly understood. Hence, this study aimed to clarify the reproducibility of PCA‐based DDG on texture features in non‑small cell lung cancer (NSCLC) patients with ^18^F‐Fluorodeoxyglucose (^18^F‐FDG) Positron emission tomography/computed tomography (PET/CT).

**Methods:**

Twenty patients with NSCLC who underwent ^18^F‐FDG PET/CT in routine clinical practice were retrospectively analyzed. Each patient's PET data were reconstructed in two PET groups of no gating (NG‐PET) and PCA‐based DDG gating (DDG‐PET). Forty‐six image features were analyzed using LIFEx software. Reproducibility was evaluated using Lin's concordance correlation coefficient ($\rho_{c}$) and percentage difference (%Diff). Non‐reproducibility was defined as having unacceptable strength $(\rho_{c}$ < 0.8) and a %Diff of >10%. NG‐PET and DDG‐PET were compared using the Wilcoxon signed‐rank test.

**Results:**

A total of 3/46 (6.5%) image features had unacceptable strength, and 9/46 (19.6%) image features had a %Diff of >10%. Significant differences between the NG‐PET and DDG‐PET groups were confirmed in only 4/46 (8.7%) of the high %Diff image features.

**Conclusion:**

Although the DDG application affected several texture features, most image features had adequate reproducibility. PCA‐based DDG‐PET can be routinely used as interchangeable images for texture feature extraction from NSCLC patients.

## 1 INTRODUCTION

Lung cancer remains the leading cause of cancer‐related death in recent years, and sustainable measures in the fight against this pathology are needed.^1^ Positron emission tomography/computed tomography (PET/CT) performed with 2‐[fluorine‐18] fluoro‐2‐deoxy‐D‐glucose (^18^F‐FDG) is an important modality for the diagnosis and management of non‑small cell lung cancer (NSCLC).^2^, ^3^, ^4^ In NSCLC, the standardized uptake value (SUV) obtained from PET images is a predictor of local recurrence and pleural invasion.^5^, ^6^, ^7^, ^8^ In the field of radiomics, specific PET texture features are associated with predictors of radiotherapy or chemoradiotherapy outcomes in NSCLC.^9^, ^10^, ^11^, ^12^, ^13^ Moreover, PET texture features have a potential for application to Erlotinib treatment response and personalized treatment strategies.^14^ However, PET image features contain technical and physical variability. Particularly, unfixed intrapulmonary tumors are affected by respiratory movements and the PET images are degraded due to unavoidable blurring.^15^, ^16^, ^17^ Xu et al. evaluated 487 radiomics features using a respiratory motion artifact and reported that only 79 (16%) features were stable.^18^

The data‐driven respiratory gating (DDG) is a novel algorithm that provides respiratory‐gated PET images.^19^, ^20^ The DDG uses internal PET respiratory data tracking that completely differs from conventional external device tracking and is thought to directly reflect the radioactivity of internal organ motion.^21^, ^22^ An automated procedure using a PET internal database enables the provision of respiratory‐gated PET images in routine clinical practice.^21^ The application of DDG improves the PET image quality and quantitative value by reducing blurring due to respiratory motion.^22^, ^23^, ^24^ In commercial PET/CT, the different motion analysis methods of the principal component analysis (PCA), spectrum analysis method (SAM), and center of mass method are used, and they provide respiratory‐gated PET images.^19^, ^25^, ^26^

To date, several studies on reproducibility comparisons between non‐gated PET and respiratory‐gated PET of image features in lung cancer have been conducted. Yip et al. evaluated the variability of texture features between static PET images and respiratory‐gated PET images from external device methods in NSCLC patients.^27^ Similarly, Oliver et al. evaluated the expanded 56‐image features and reported the variability.^28^ On the contrary, Grootjans et al. reported that respiratory gating did not result in statistically significant differences in textural parameters between non‐gated PET and respiratory‐gated PET in the whole cohort.^29^ Faist et al. evaluated the reproducibility of image features extracted from SAM‐based DDG and reported that most radiomics features had high reproducibility^30^ The interpretation of previous results requires further evaluation. Additionally, the reproducibility of PCA‐based DDG has not been evaluated. The standardization of PET images is desired to not limit the usefulness and application of texture analysis in radiomics.^13^, ^31^, ^32^ In this study, we evaluated the reproducibility of the PCA‐based DDG on texture features in NSCLC patients with ^18^F‐FDG PET/CT.

## 2 METHODS

### 2.1 Patients

Twenty NSCLC patients who underwent ^18^F‐FDG PET/CT in routine clinical practice were retrospectively analyzed. Each patient had a single intrapulmonary lesion that was selected per the 10−64‐mm size criterion and the 2.5 > SUV criterion.^5^, ^30^ Our institutional ethics committee approved this study (No. 2021‐GB‐029).

### 2.2 PET/CT scan

The Discovery MI (GE Healthcare, Milwaukee, WI, USA) PET/CT system, which is composed of the silicon photomultiplier (SiPM) digital PET scanner and the 64‐slice helical CT scanner, was used. The PET scanner included four blocks of cerium‐doped lutetium yttrium orthosilicate digital detector (Light‐Burst Detector), and time‐of‐flight PET system. All patients fasted for more than 6 h before ^18^F‐FDG (4 MBq/kg) was injected. After a 60‐min uptake period, free‐breathing helical CT scanning and free‐breathing PET scanning were conducted. The scanning CT parameters were set at 120 kV voltage with autoexposure control, 3.75 mm slice thickness, and 512 × 512 matrix. The PET scans were conducted in a 256 × 256 matrix with 2 min (body mass index: BMI <22) or 2.5 min (BMI ≧22) list mode acquisition per bed position. The DDG application, Advanced Motion Free, (AMF; GE Healthcare, Milwaukee, WI, USA) provided respiratory gating PET images based on the PCA. The AMF acceptance was set at an *R*‐value of >15, 30% offset, and 50% width value.^23^ Only the lung segment required a PET scan duration that was twice as long as that required for AMF.

### 2.3 Image reconstruction

All PET images were reconstructed using the Bayesian penalized likelihood reconstruction algorithm, Q.Clear (GE Healthcare, Milwaukee, WI, USA), with a β value of 500. The Q.Clear algorithm included the point spread function modeling and the penalty noise control algorithm. Model‐based scatter correction with Compton‐scattering recovery and CT‐based attenuation correction methods were applied. In our study, there were two PET dataset groups, no gating (NG‐PET) and applied DDG gating (DDG‐PET), from each NSCLC patient.

### 2.4 Texture analysis

The texture analysis was conducted using the LIFEx software program.^33^ LIFEx is a free Java application that enables radiomic feature calculation for the characterization of tumor heterogeneity in multimodality imaging. All patient's PET images were post‐processed through conversion to 2‐mm isotropic voxels and 64‐range intensity discretization.^32^ A volume of interest (VOI) with a threshold value of 40% maximum SUV (VOI_40%_) was placed on each NSCLC lesion. The VOI_40%_, which had excellent inter‐operator reproducibility of texture features, was recommended for heterogeneity determination of lesions affected by respiratory movements.^34^ The following 46 image features were calculated for each NSCLC lesion: three quantitative indices (SUVmean, SUVmax, and total lesion glycolysis); six histogram features (Skewness, Kurtosis, Excess kurtosis, Entropy_log10, Entropy_log2, and Energy); five shape features (Volume (mL), Volume (voxel), Sphericity, Surface (mm^2^), and Compacity); eighteen second‐order parameters included in the Gray‐Level Co‐occurrence Matrix (GLCM) and the Gray‐Level Run‐Length Matrix (GLRLM); fourteen higher‐order parameters included in the Neighborhood Gray‐Level Different Matrix (NGLDM), and the Gray‐Level Zone Length Matrix (GLZLM). All PET image features were extracted as absolute values of continuous variables and treated as non‐normally distributions due to the limited number of patients.

### 2.5 Data analysis

Lin's concordance correlation coefficient ($\rho_{c}$) was calculated for all image features across NG‐PET and DDG‐PET to determine the correlation between two the PET groups (Equation 1).^35^ Also, the %Diff was calculated for all image features across NG‐PET and DDG‐PET to determine the variability between the two PET groups (Equation 2).

(1) $$\rho_{c}=\frac{2\rho \sigma_{x}\sigma_{y}}{\sigma_{x}^{2}+\sigma_{y}^{2}+{\left(\mu_{x}-\mu_{y}\right)}^{2}}$$

(2) $$\%Diff=\frac{\left(\mu_{y}-\mu_{x}\right)}{\mu_{x}}\times 100$$

where $\sigma_{x}$ and $\sigma_{y}$ are the standard deviations of each image feature for NG‐PET and DDG‐PET, respectively, $\mu_{x}$ and $\mu_{y}$ are the means (Equation 1) and medians (Equation 2) of each image feature for NG‐PET and DDG‐PET, respectively, and ρ is the coefficient of the correlation between NG‐PET and DDG‐PET. Agreement was determined as follows: high strength of agreement ($\rho_{c}\;$> 0.99); substantial strength of agreement ($\rho_{c}$: 0.95 to 0.99); moderate strength of agreement $(\rho_{c}$: 0.90 to 0.95); poor strength of agreement ($\rho_{c}$: 0.80 to 0.90); unacceptable strength ($\rho_{c}$ < 0.80). Non‐reproducible image features were defined as those with unacceptable strength of agreement ($\rho_{c}$ < 0.80) and a %Diff of >10%.^30^

### 2.6 Statistical analysis

Continuous variables are expressed as median values and interquartile ranges (IQR; 25 to 75 percentile). All statistical analyses were performed using EZR (Saitama Medical Center, Jichi Medical University, Saitama, Japan), a graphical user interface for R (The R Foundation for Statistical Computing, Vienna, Austria), and Statistics Toolkit (Chinese University of Hong Kong, Shatin, Hong Kong).^36^ Comparisons between NG‐PET and DDG‐PET were performed using the Wilcoxon signed‐rank test. *p*‐values less than 0.05 were considered statistically significant.

## 3 RESULTS

### 3.1 Patients with NSCLC

**FIGURE 1:**
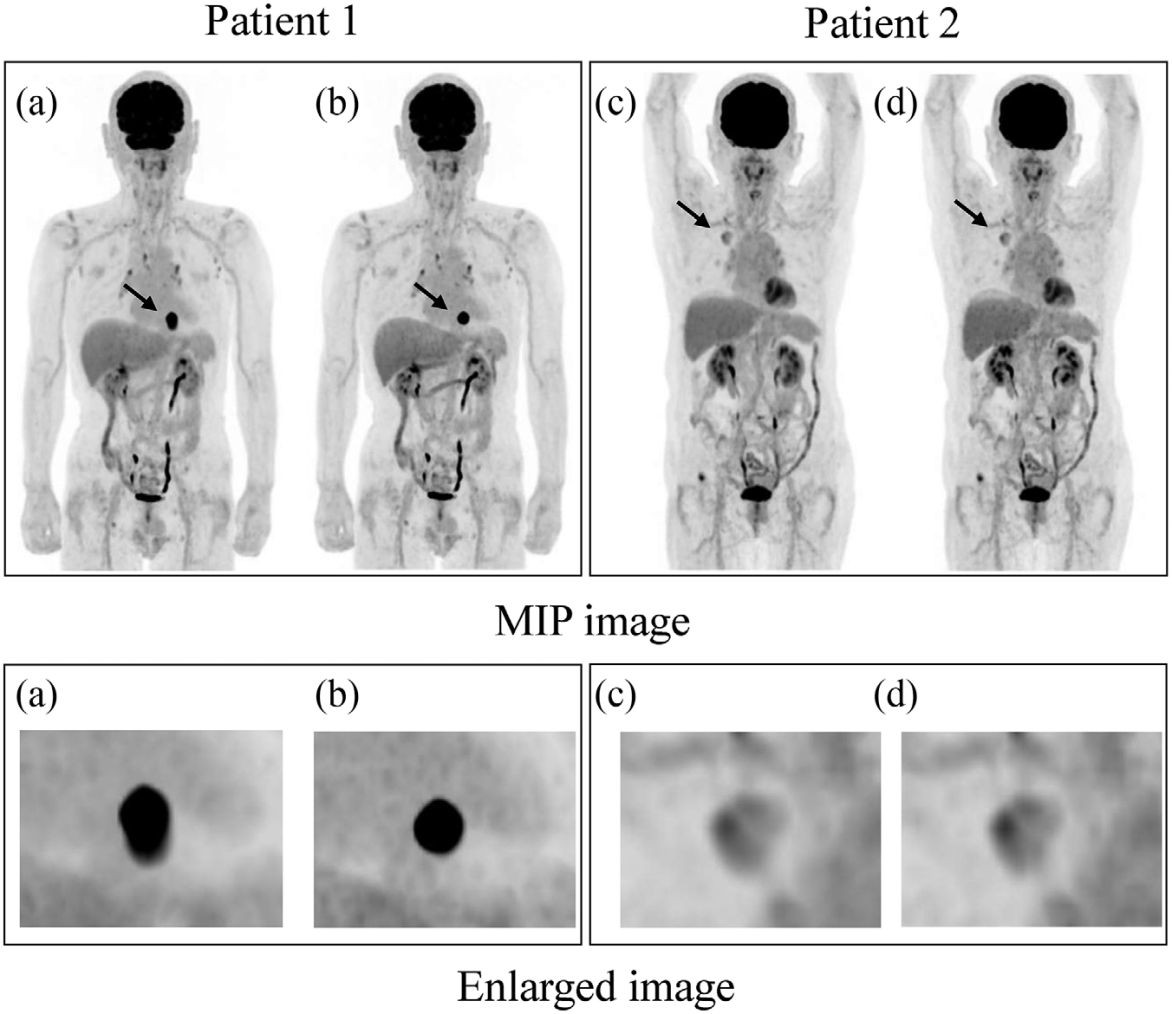
Examples of analyzed NSCLC lesions, Representative PET images taken from two patients show the deformation of metabolic shape (patient 1) and change in heterogeneity (patient 2) due to DDG‐PET application. Patient 1: Squamous cell carcinoma of left lower lobe. Patient 2: Adenocarcinoma of right upper lobe. Upper: PET maximum intensity projection (MIP) image. Lower: enlarged NSCLC lesions. (a and c) Without motion correction. (b and d) With DDG‐PET application. The display color scale was set to invert gray, and identical display density.

Table 1 shows the characteristics of NSCLC patients, while Figure 1 shows the examples of analyzed NSCLC lesions in two PET groups. The 20 NSCLC lesions were located in 11 superior lobes, 1 middle lobe, and 8 inferior lobes. The CT‐based mean tumor size was 28.2 mm, and the histopathology results indicated a diagnosis of adenocarcinoma and squamous cell carcinoma. The initial staging of lung cancer classified patients as ten in stage I, one in stage II, two in stage III, and seven in stage IV.

**TABLE 1:** Characteristics of NSCLC. patients

Characteristics	
Mean age (y)	69.2
Mean tumor size (mm)	28.2
**Gender**	
Male	12
Female	8
**Histology**	
Adenocarcinoma	15
Squamous cell carcinoma	5
**Location**	
Superior lobes (right)	10
Superior lobes (left)	1
Middle lobes (right)	1
Inferior lobes (right)	4
Inferior lobes (left)	4
**Purpose**	
Initial staging	18
Monitoring of chemotherapy	2
**Stage**	
IA	7
IB	3
IIB	1
IIIB	2
IVA	4
IVB	3

### 3.2 Lin's concordance correlation coefficient

Table 2 shows the reproducibility of Lin's concordance correlation coefficient. There was high strength of agreement in 5/46 (10.9%) features, substantial strength of agreement in 16/46 (34.8%) features, moderate strength of agreement in 14/46 (30.4%) features, poor strength of agreement in 8/46 (17.4%) features, and unacceptable strength of agreement in 3/46 (6.5%) features, respectively. The unacceptable strength feature is identified in the long‐zone emphasis, long‐zone low gray‐level emphasis, and long‐zone high gray‐level emphasis of GLZLM.

**TABLE 2:** Lin's concordance correlation coefficient.

Image features	Lin's concordance correlation coefficient	Level of agreement
**Quantitative**		
SUVmean	0.97	Substantial strength
SUVmax	0.96	Substantial strength
Total lesion glycolysis (TLG)	1.00	High strength
**First Order**		
HISTO_Skewness	0.85	Poor strength
HISTO_Kurtosis	0.82	Poor strength
HISTO_Excess kurtosis	0.82	Poor strength
HISTO_Entropy_log10	0.97	Substantial strength
HISTO_Entropy_log2	0.97	Substantial strength
HISTO_Energy	0.97	Substantial strength
SHAPE_Volume (mL)	0.99	High strength
SHAPE_Volume (voxel)	0.99	High strength
SHAPE_Sphericity	0.95	Moderate strength
SHAPE_Surface (mm^2^)	0.99	High strength
SHAPE_Compacity	0.98	Substantial strength
**GLCM**		
Homogeneity	0.94	Moderate strength
Energy	0.94	Moderate strength
Contrast	0.91	Moderate strength
Correlation	0.91	Moderate strength
Entropy_log10	0.99	Substantial strength
Entropy_log2	0.99	Substantial strength
Dissimilarity	0.94	Moderate strength
**GLRLM**		
Short‐run emphasis (SRE)	0.94	Moderate strength
Long‐run emphasis (LRE)	0.92	Moderate strength
Low gray‐level run emphasis (LGRE)	0.94	Moderate strength
High gray‐level run emphasis (HGRE)	0.96	Substantial strength
Short‐run low gray‐level emphasis (SRLGE)	0.92	Moderate strength
Short‐run high gray‐level emphasis (SRHGE)	0.97	Substantial strength
Long‐run low gray‐level emphasis (LRLGE)	0.96	Substantial strength
Long‐run high gray‐level emphasis (LRHGE)	0.85	Poor strength
Gray‐level non‐uniformity for run (GLNU)	0.96	Substantial strength
Run length non‐uniformity (RLNU)	1.00	High strength
Run percentage (RP)	0.90	Moderate strength
**NGLDM**		
Coarseness	0.94	Moderate strength
Contrast	0.90	Poor strength
Busyness	0.81	Poor strength
**GLZLM**		
Short‐zone emphasis (SZE)	0.86	Poor strength
Long‐zone emphasis (LZE)	**0.67**	Unacceptable strength
Low gray‐level zone emphasis (LGZE)	0.93	Moderate strength
High gray‐level zone emphasis (HGZE)	0.97	Substantial strength
Short‐zone low gray‐level emphasis (SZLGE)	0.86	Poor strength
Short‐zone high gray‐level emphasis (SZHGE)	0.96	Substantial strength
Long‐zone low gray‐level emphasis (LZLGE)	**0.60**	Unacceptable strength
Long‐zone high gray‐level emphasis (LZHGE)	**0.71**	Unacceptable strength
Gray‐level non‐uniformity for zone (GLNU)	0.97	Substantial strength
Zone length non‐uniformity (ZLNU)	0.99	Substantial strength
Zone percentage (ZP)	0.91	Moderate strength

**Bold**: Non‐reproducible.

### 3.3 Percentage difference

Table 3 shows the percentage difference. A %Diff of >10% was identified in 9/46 (19.6%) image features, and a significant difference was observed in 4/46 (8.7%) image features. The significantly high %Diff was the gray‐level nonuniformity for the run (GLNU) and run‐length nonuniformity (RLNU) of GLRLM, Busyness of NGLDM, and the short‐zone high gray‐level emphasis of GLZLM.

**TABLE 3:** Percentage difference.

Image features	NG‐PET		DDG‐PET		**%Diff**	***p‐* values**
**Quantitative**	**Median**	**IQR**	**Median**	**IQR**		
SUVmean	6.80	3.00–8.34	6.92	3.83–8.49	1.65	<0.001*
SUVmax	10.65	5.10–13.60	10.88	6.37–13.98	2.16	<0.001*
Total lesion glycolysis (TLG)	27.79	6.47–59.99	25.62	6.25–58.25	‐7.83	0.083
**First Order**						
HISTO_Skewness	1.68	0.99–3.15	1.69	1.18–3.42	0.73	0.48
HISTO_Kurtosis	4.92	2.37–12.93	5.67	2.86–16.15	15.14	0.50
HISTO_Excess kurtosis	1.92	−0.63–9.93	2.67	−0.14–13.15	38.75	0.50
HISTO_Entropy_log10	1.34	0.97–1.39	1.32	1.05–1.39	−1.29	0.37
HISTO_Entropy_log2	4.44	3.22–4.60	4.38	3.48–4.61	−1.29	0.37
HISTO_Energy	0.05	0.05–0.13	0.05	0.05–0.11	−0.63	0.67
SHAPE_Volume (mL)	4.69	1.25–7.93	4.50	1.18–7.24	−3.92	<0.001*
SHAPE_Volume (voxel)	586.00	155.75–990.75	563.00	147.00–904.50	−3.92	<0.001*
SHAPE_Sphericity	0.82	0.77–0.85	0.82	0.76–0.88	−0.56	1.000
SHAPE_Surface (mm^2^)	1692.57	660.71–2400.53	1695.66	608.92–2294.44	0.18	0.002*
SHAPE_Compacity	2.68	1.93–3.29	2.68	1.93–3.18	0.00	0.001*
**GLCM**						
Homogeneity	0.34	0.26–0.48	0.34	0.27–0.45	−0.72	0.35
Energy	0.01	0.00–0.02	0.01	0.00–0.02	9.51	0.76
Contrast	20.64	5.69–50.35	21.65	7.10–48.97	4.87	0.070
Correlation	0.61	0.43–0.65	0.57	0.43–0.64	−7.12	0.007*
Entropy_log10	2.10	1.84–2.52	2.12	1.85–2.45	1.15	0.81
Entropy_log2	6.97	6.11–8.36	7.05	6.13–8.14	1.15	0.81
Dissimilarity	3.54	1.84–5.70	3.55	2.07–5.61	0.29	0.070
**GLRLM**						
Short‐run emphasis (SRE)	0.94	0.90–0.96	0.94	0.91–0.96	−0.38	0.57
Long‐run emphasis (LRE)	1.28	1.18–1.46	1.29	1.19–1.50	1.42	0.67
Low gray‐level run emphasis (LGRE)	0.00	0.00–0.01	0.00	0.00–0.01	−8.27	0.005*
High gray‐level run emphasis (HGRE)	523.65	109.77–769.05	534.13	174.95–792.82	2.00	0.001*
Short‐run low gray‐level emphasis (SRLGE)	0.00	0.00–0.01	0.00	0.00–0.01	−9.07	0.002*
Short‐run high gray‐level emphasis (SRHGE)	497.12	99.54–716.38	496.19	157.58–742.50	−0.19	<0.001*
Long‐run low gray‐level emphasis (LRLGE)	0.00	0.00–0.02	0.00	0.00–0.01	−0.27	0.002*
Long‐run high gray‐level emphasis (LRHGE)	626.47	170.60–1024.57	664.89	233.96–1057.68	6.13	0.006*
Gray‐level non‐uniformity for run (GLNU)	29.88	14.08–52.13	26.26	14.01–50.75	**−12.12**	0.015*
Run length non‐uniformity (RLNU)	451.96	133.25–742.85	392.39	127.29–696.58	**−13.18**	<0.001*
Run percentage (RP)	0.92	0.88–0.95	0.92	0.87–0.94	−0.53	0.57
**NGLDM**						
Coarseness	0.01	0.01–0.04	0.01	0.01–0.04	−4.07	0.55
Contrast	0.17	0.11–0.29	0.17	0.10–0.34	1.09	0.43
Busyness	0.21	0.09–0.71	0.13	0.08–0.50	**−37.28**	0.006*
**GLZLM**						
Short‐zone emphasis (SZE)	0.59	0.45–0.69	0.63	0.50–0.71	6.86	0.012*
Long‐zone emphasis (LZE)	25.89	9.08–147.20	26.43	8.58–141.23	2.08	0.25
Low gray‐level zone emphasis (LGZE)	0.00	0.00–0.01	0.00	0.00–0.01	−5.55	0.007*
High gray‐level zone emphasis (HGZE)	445.56	101.08–622.41	445.33	148.53–685.93	−0.05	0.003*
Short‐zone low gray‐level emphasis (SZLGE)	0.00	0.00–0.01	0.00	0.00–0.00	−17.32	0.12
Short‐zone high gray‐level emphasis (SZHGE)	246.40	43.12–382.73	292.39	68.54–460.53	**18.67**	0.002*
Long‐zone low gray‐level emphasis (LZLGE)	0.06	0.01–4.14	0.06	0.01–1.75	−7.58	0.15
Long‐zone high gray‐level emphasis (LZHGE)	16159.34	7056.12–34578.40	20941.71	3687.14–40876.91	29.60	0.33
Gray‐level non‐uniformity for zone (GLNU)	8.98	4.27–14.25	9.18	4.19–16.90	2.24	0.96
Zone length non‐uniformity (ZLNU)	41.56	10.68–155.70	45.96	17.74–134.37	10.59	0.87
Zone percentage (ZP)	0.36	0.13–0.51	0.36	0.15–0.54	0.10	0.13

**Bold**: Non‐reproducible.

^*^Statistically significant.

## 4 DISCUSSION

Multifaceted validation is required to pursue the potential of radiomics in lung cancer.^13^, ^31^, ^32^ The DDG‐PET respiratory gating contributes to the improvement of image quality; however, it may affect the extracted image features.^22^, ^23^, ^24^, ^37^, ^38^ Although several studies have demonstrated the reproducibility or variability between non‐gated PET and external respiratory‐gated PET methods, there are few studies on the novel DDG‐based respiratory gating.^27^, ^28^, ^29^, ^30^ To the best of our knowledge, this is the first study to evaluate the reproducibility of PCA‐based DDG on texture features in patients with NSCLC.

In this study, total of 39/46 (84.8%) image features demonstrated repeatability based on adequate strength of agreement and non‐significant variability. The reason for this is thought to be associated with the range surrounded by the centered 40% stable VOI.^34^ As a result, each patient's NSCLC lesion was not significantly affected by the blurring reduction due to the application of DDG. The VOI setting for tumor and process of intensity conversion are considered to have effect on the PET image features, while the VOI_40%_ and 64‐range intensity discretization process adopted in this study are standard and common.^13^, ^31^, ^34^ The reproducibility rate of this study was as high as the SAM‐based DDG‐PET study that 131/141 (92.9%) image features had reproducibility.^30^ The similar high reproducibility of PCA‐based DDG‐PET and SAM‐based DDG‐PET suggests the potential for interchangeability of texture features between other DDG‐PET methods and NG‐PET. In contrast, some texture features associated with GLRLM and GLZLM were not reproducible because these matrix indices, which are known to be highly variable, are calculated from large scales with many voxels.^39^, ^40^ In other words, non‐reproducible features are thought to be affected by blurring reduction due to the application of DDG. From a different perspective, reproducibility confirmed in useful texture features such as GLCM Entropy and GLCM Dissimilarity is important.^9^, ^10^, ^11^ High reproducibility based on GLCM was also reported in a previous study.^32^ In summary, our findings demonstrate that PCA‐based DDG‐PET images in routine clinical practice can be considered to have compatible texture features with NG‐PET images, except for several specific features in NSCLC.

However, this study has several limitations. First, free‐breathing CT was used for attenuation correction. The CT images used for attenuation correction have been reported to affect the quality of DDG‐PET images and may also affect the image features.^41^, ^42^ Second, although we used a respiratory motion criterion with an *R*‐value of ≥15, it was difficult to pursue information on the absolute amount of tumor movement and the accompanying blurring. Third, the image analysis was restricted to the tumor size criterion of ≥10 mm. In general, small lesions are difficult to evaluate via PET/CT because they are greatly affected by respiratory movements and have partial volume effects.^43^, ^44^, ^45^ Fukai et al. reported that PCA‐based DDG was also applied to sub‐centimeter small lesions measuring >6 mm.^46^ Finally, this study was conducted in limited number of patients, and a single‐machine and single‐center study. The subdivided groups such as lesion location have not been evaluated. Although this study design can reduce machine bias, it entails the possibility of the inherent performance of equipment affecting the results. Despite these limitations, our study provides important information about the repeatability and interchangeability of PCA‐based DDG‐PET texture features for radiomics. Further studies are required for the development of radiomics in patients with NSCLC.

## 5 CONCLUSION

Although the DDG application affected several texture features that were calculated from large‐scale voxel ranges, most texture features had adequate reproducibility. The PCA‐based DDG‐PET can be routinely used as a source of interchangeable images for texture feature extraction in patients with NSCLC.

## AUTHOR CONTRIBUTIONS

All authors contributed to the study design and data interpretation. Shohei Fukai and Hiromitsu Daisaki wrote the article and contributed to the entire study procedure as the principal investigators. Naoki Shimada, Takuro Umeda, Kazuki Motegi, and Ryoma Ito contributed to image analyses. Mitsutomi Ishiyama and Takashi Terauchi contributed to the obtention of ethical approval for this study.

## CONFLICT OF INTEREST STATEMENT

The authors declare no conflicts of interest associated with this study.
